# Maximum inspiratory pressure as a clinically meaningful trial endpoint for neuromuscular diseases: a comprehensive review of the literature

**DOI:** 10.1186/s13023-017-0598-0

**Published:** 2017-03-16

**Authors:** Benedikt Schoser, Edward Fong, Tarekegn Geberhiwot, Derralynn Hughes, John T. Kissel, Shyam C. Madathil, David Orlikowski, Michael I. Polkey, Mark Roberts, Harm A. W. M. Tiddens, Peter Young

**Affiliations:** 1 Friedrich-Baur-Institute, Department of Neurology, Ludwig-Maximilian University, Ziemssenstr. 1a, D-80336 Munich, Germany; 2 Division of Pediatric Pulmonology, Asthma, and Sleep Medicine, Stanford University, Stanford, USA; 3 University Hospitals Birmingham, Selly Oak Hospital, Birmingham, UK; 4 Department of Haematology, Royal Free & University College Medical School, London, UK; 5 Department of Neuroscience, The Ohio State University Wexner Medical Center, Columbus, OH USA; 6 Department of Respiratory Medicine, University Hospital Birmingham NHS Foundation Trust, Queen Elizabeth Hospital, Birmingham, England UK; 7 Intensive Care Unit and Home Ventilation Unit, Raymond Poincare Teaching Hospital, Garches, France; 8 NIHR Respiratory Biomedical Research Unit of the Royal Brompton and Harefield NHS Foundation Trust and Imperial College London, London, UK; 9 Greater Manchester Neurosciences Unit, Salford Royal NHS Foundation Trust, Salford, UK; 10 Department of Pediatric Pulmonology and Allergology, Sophia Children’s Hospital, Rotterdam, The Netherlands; 11 Department of Sleep Medicine and Neuromuscular Disorders, University of Münster, Münster, Germany

**Keywords:** Maximum inspiratory pressure, Neuromuscular disease, Respiratory failure, Endpoint, Survival, Pulmonary function testing, Spirometry

## Abstract

Respiratory muscle strength is a proven predictor of long-term outcome of neuromuscular disease (NMD), including amyotrophic lateral sclerosis, Duchenne muscular dystrophy, and spinal muscular atrophy. Maximal inspiratory pressure (MIP), a sensitive measure of respiratory muscle strength, one of several useful tests of respiratory muscle strength, is gaining interest as a therapeutic clinical trial endpoint for NMD. In this comprehensive review we investigate the use of MIP as a measure of respiratory muscle strength in clinical trials of therapeutics targeting respiratory muscle, examine the correlation of MIP with survival, quality of life, and other measures of pulmonary function, and outline the role of MIP as a clinically significantly meaningful outcome measure. Our analysis supports the utility of MIP for the early evaluation of respiratory muscle strength, especially of the diaphragm, in patients with NMD and as a surrogate endpoint in clinical trials of therapies for NMD.

## Background

Weakness of the respiratory muscles is especially common among patients who have an acute or chronic neuromuscular disease (NMD), including amyotrophic lateral sclerosis (ALS), Guillain-Barré syndrome, spinal muscular atrophy, myotonic dystrophy type 1, Duchenne and other muscular dystrophies, and Pompe disease [1–3]. In patients with a NMD, irrespective of age, pathophysiological mechanisms that lead to the development of respiratory failure frequently include progressive weakness in the inspiratory muscles, predominantly the diaphragm, as indicated by respiratory patterns with low tidal volumes [4, 5]. However, the etiology of respiratory dysfunction can vary somewhat between different conditions [6–10].

NMD may impact different facets of respiratory muscle function (inspiratory, expiratory extrathoracic airways) to different extents. While expiratory muscle weakness is associated with ineffective cough, inspiratory muscle weakness causes dyspnea and/or nocturnal alveolar hypoventilation [4]. Dyspnea, which results in an increased sense of effort, is a subjective sensation of breathing discomfort, likened to being smothered or suffocated. Nocturnal hypoventilation disrupts normal sleep architecture, initially in REM sleep leading to excessive daytime fatigue and morning headaches due to hypercapnia [11, 12]. Other symptoms of nocturnal hypoventilation, include insomnia, hypersomnolence, or impaired cognition. In patients with NMD, hypoventilation during REM sleep may be an early marker of the functional impact of diaphragm weakness [12]. Lastly, expiratory muscle weakness leads to ineffective clearance of airway secretions, and depending on the severity of the muscle weakness, these patients are thus at a higher risk for aspiration (more so since such patients have concomitant swallowing difficulties), bronchitis, and pneumonia [3, 11].

The symptoms of respiratory muscle weakness can infringe significantly on daily activities (eg, walking and eating) and quality of life (QoL) of patients with NMD. Respiratory muscle weakness ultimately leads to respiratory failure and early mortality [13, 14] or the need for noninvasive or invasive mechanical ventilation to prolong survival [3, 13, 15–18]. Indeed, respiratory failure secondary to muscle weakness is a common cause of premature death in NMD [19]. It is, therefore, critical that patients with NMD are regularly monitored (including for measures of respiratory muscle strength and function, cough, and swallowing [20, 21]) and subsequently managed accordingly.

Maximal inspiratory pressure (MIP) and maximal expiratory pressure (MEP) are direct measures of respiratory muscle strength and may be more sensitive in detecting early respiratory muscle dysfunction compared with spirometry, but MIP and MEP are not usually performed on all patients referred for PFTs [22]. MIP and MEP are noninvasive, straightforward tests in which individuals are asked to perform a forceful inspiration after an expiration to residual volume level (in the case of MIP) or expiration after a full inspiration to total lung capacity (TLC; in the case of MEP) with an open glottis against an occluded mouthpiece [5, 22, 23]; the tests are generally practical in individuals older than 6 or 7 years of age. They are indicated if muscle weakness could be contributing to abnormal spirometry test results, such as a low vital capacity (VC) [22]. MIP is a measure of global inspiratory muscle strength and therefore has a close relationship with diaphragmatic strength, since the diaphragm is the major inspiratory muscle; MEP is generated through the abdominal and intercostal muscles [23–26].

Some debate exists about a “normal” value of MIP and MEP, and different cut-off points for percentages of predicted values have been recommended, not taking into consideration any adjustments other than gender; a recent study [27] has recommended using more ‘cautious’ reference equations. A 2009-update [22] of the statement on respiratory muscle testing by the American Thoracic Society and the European Respiratory Society [23] on the assessment of MRPs, provides more exact estimates for normal values of MIP and MEP and recommends the use of a flanged mouthpiece for the measurements. Furthermore, low MIPs can sometimes be difficult to interpret in patients with advanced illness because exertion of maximal effort is a challenge for these patients.

Together, MIP and MEP measurements can accurately assess respiratory muscle weakness, and MIP may even predict diaphragm weakness before a significant change in spirometry endpoints (eg, forced vital capacity [FVC]) [28]. However, despite the potential advantages of MIP and MEP, respiratory muscle function should be evaluated with the complete array of widely available lung volume and pressure measurements, rather than relying upon individual measurements used in isolation [29].

Given that respiratory muscle dysfunction—especially that of the diaphragm—is common to NMDs, then measurement of respiratory muscle strength through MIP may provide an additional meaningful endpoint in trials of therapeutics targeting respiratory muscle in patients with NMDs. With this in mind, we examined the use of MIP as a clinical endpoint in trials of therapeutics and investigated the relationship of MIP values with other parameters associated with respiratory muscle dysfunction, including survival and QoL metrics.

## Methods

We conducted literature searches in multiple databases (EMBASE, Scopus, PubMed, ProQuest, and Google Scholar; articles published before August 2015) and included clinical studies that used MIP as either a primary or secondary clinical endpoint outcome measure and reported a relationship between MIP and survival, QoL, pulmonary, and/or nonpulmonary functional endpoints. BioMarin Pharmaceutical Inc. provided funding for this analysis and for medical writing and editorial support during manuscript development. BioMarin Pharmaceutical Inc. was not involved in the collection, analysis, or interpretation of data. All authors had full access to study data and were solely responsible for the decision to submit for publication.

Search terms included the following key words: (“maximal inspiratory pressure” or “nasal inspiratory pressure” or “negative inspiratory force” or “MIP” or “PImax”) AND (“outcome” or “endpoint” or “efficacy” or “treatment effectiveness” or “sleep-disordered breathing” or “nocturnal hypoventilation” or “mortality” or “survival” or “death”). We initially screened literature records on the basis of title and abstract and excluded records not meeting the inclusion criteria (eg, no MIP results reported, disease and/or natural history focused).

To identify open, ongoing clinical trials that are using MIP as an endpoint (primary or secondary), we searched the ClinicalTrials.gov database (www.clinicaltrials.gov). The search was conducted on July 12, 2015; search terms included *maximal inspiratory pressure*, *PImax*, *and negative inspiratory force*.

## Results

### MIP as an endpoint in clinical trials

We identified 8 publications in which MIP was used as a primary or secondary endpoint in a randomized controlled trial (RCT) of a pharmacologic therapy in a broad spectrum of conditions (Table 1) [26, 30–36]. One of these studies was in patients with Duchenne muscular dystrophy [36]. Across the studies, the MIP endpoint was reported to be a sensitive and specific clinical measure for evaluation of the pharmacologic interventions, all of which directly targeted the respiratory musculature.

**Table 1:** Completed RCTs using MIP as a clinical endpoint

Reference	MIP Endpoint	Treatment Group	N	MIP Result
Golparvar M et al., 2005 [30]	Primary	Progesterone administration in adult trauma patients during partial support ventilation	40	MIP significantly increased (*P* < 0.05) 3 h after administration
Gontijo-Amaral C et al., 2012 [26]	Primary	Oral magnesium supplementation in adolescent cystic fibrosis patients	44	Significant increase in MIP between intervention and placebo period (*P* < 0.001)
Mackersie RC et al., 1991 [31]	Primary	Continuous epidural or continual IV infusions of fentanyl in patients with multiple rib fractures	32	Significant increase in MIP in epidural and fentanyl epidural groups compared with pre-analgesia (*P* < 0.05)
Sosis M et al., 1987 [32]	Primary	Atracurium treatment in patients requiring intubation	39	Significant decrease in MIP in patients receiving atracurium compared with placebo (*P* < 0.05)
Andreas S et al., 2006 [35]	Primary	Irbesartan treatment in COPD patients	60	No significant difference in MIP after 4 months of treatment (*P* = 0.16). Reporting a large standard deviation of MIP.
Skorodin MS et al., 1995 [33]	Primary	Magnesium sulphate administration in COPD patients	72	No significant difference in MIP after 20 and 45 min of treatment (*P* = NS)
Weisberg J et al., 2002 [34]	Primary	Megestrol acetate administration in COPD patients	128	No significant difference in MIP (*P* = NS). Reporting a large standard deviation of MIP.
Buyse GM et al., 2013 [36]	Secondary	Idebenone treatment in Duchenne muscular dystrophy patients	21	MIP improved in idebenone group but deteriorated in placebo group (*P* = NS)

*COPD* chronic obstructive pulmonary disease, *IV* intravenous, *MIP* maximum inspiratory pressure, *NS* not significant

A search of the clinical trials database revealed 31 open trials where MIP was cited as either a primary or secondary endpoint (Table 2). The trials reflected a range of clinical conditions; approximately 1 in 3 were in individuals with a NMD (including ALS, Duchenne muscular dystrophy, myasthenia gravis, Pompe disease, and X-linked myotubular myopathy). This suggests that MIP is gaining momentum as clinical endpoint for monitoring respiratory muscle function in these patients.

**Table 2:** Ongoing^a^ clinical trials with MIP as an endpoint

NCT number	Study Title	Study Design	Primary or Secondary Endpoint	Estimated Study Completion Date	Estimated Enrollment
Neuromuscular diseases					
Amyotrophic lateral sclerosis					
NCT02478450	Study to Investigate the Safety of the Transplantation (by Injection) of Human Glial Restricted Progenitor Cells (hGRPs; Q-Cells®) Into Subjects With Amyotrophic Lateral Sclerosis (ALS)	Nonrandomized, open-label, parallel group study	Secondary	December 2017	12
Duchenne muscular dystrophy					
NCT02310906	Phase I/II Study of SRP-4053 in DMD Patients	Randomized, placebo-controlled, double-blind, parallel group study	Secondary	December 2016	48
NCT01999075	Stacking Exercises Aid the Decline in FVC and Sick Time (STEADFAST)	Randomized, single-blind, parallel group study	Secondary	August 2016	110
NCT02255552	Confirmatory Study of Eteplirsen in DMD Patients (PROMOVI)	Nonrandomized, open-label, parallel group study	Secondary	May 2016	160
Myasthenia gravis					
NCT01047761	Exercise for Stable Myasthenia Gravis	Nonrandomized, open-label, single group study	Secondary	December 2015	30
Parkinson’s disease					
NCT02202057	Respiratory Load Magnitude Estimation in PD	Prospective, case control study	Secondary	August 2015	80
Pompe disease					
NCT02357225	A Pilot Study of Pyridostigmine in Pompe Disease	Nonrandomized, open-label, single group study	Primary	June 2017	16
NCT02354651	Response to Diaphragmatic Pacing in Subjects With Pompe Disease	Prospective, observational study	Secondary	February 2017	12
NCT01924845	BMN 701 Phase 3 in rhGAA Exposed Subjects With Late Onset Pompe Disease (INSPIRE Study)	Nonrandomized, open-label, single group study	Primary	June 2020	50
Pulmonary hypertension					
NCT02288442	Whole Muscle Exercise Training (WHOLEi + 12) in Pulmonary Hypertension	Randomized, single-blind, parallel group study	Secondary	September 2016	20
Sarcopenia					
NCT02120586	Preventive Physiotherapy Intervention in Elderly People With Sarcopenia	Randomized, single-blind, parallel group study	Primary	July 2015	70
X-linked myotubular myopathy					
NCT02453152	Respiratory Muscle Function in Untreated X-Linked Myotubular Myopathy (XLMTM)	Prospective, observational study	Secondary	December 2016	12
Other disorders (non-NMDs)					
Back pain					
NCT02429752	Low Back Pain and Breathing Pattern Dysfunction (LBP & BPD)	Open-label, single group study	Secondary	June 2016	75
Breast disease					
NCT02491762	The Effect of Breast Reconstruction Surgery Using Tissue Expanders on Respiratory Functions	Nonrandomized, open-label, parallel group study	Primary	August 2017	45
NCT02165696	Compression Bandaging and Manual Lymph Drainage in Women With Lymphedema (LYMPHATIC)	Randomized, single-blind, parallel group study	Secondary	December 2015	44
COPD					
NCT01655199	Sensitivity of the Step Test to Detect Improvement in Dyspnea Following Bronchodilation in Patients With Chronic Obstructive Pulmonary Disease (CODEx)	Double-blind, single group study	Secondary	July 2014	40
NCT01903772	Effects of Inspiratory Muscle Training in Chronic Obstructive Pulmonary Disease (COPD) (IMTCO)	Randomized, double-blind, parallel group study	Secondary	December 2015	72
NCT01956565	Feasibility of Inspiratory Muscle Training in People With COPD Who Decline Pulmonary Rehabilitation	Nonrandomized, open-label, single group study	Secondary	November 2015	20
NCT02392715	Inspiratory Muscle Training Combined With General Exercise Training in COPD (IMTGET)	Randomized, double-blind, parallel group study	Secondary	October 2016	80
NCT02007772	Effectiveness of TNI vs. BiPAP in Chronic Global Insufficiency in COPD Patients (TIBICO)	Randomized, open-label, crossover study	Secondary	August 2015	85
NCT01582958	The Effect of OMT on Patients With COPD: Correlating Pulmonary Function Tests With Biochemical Alterations	Randomized, single-blind, parallel group study	Primary	August 2015	60
NCT01037387	Effect of Noninvasive Ventilation on Physical Activity and Inflammation in COPD Patients	Randomized, open-label, parallel group study	Secondary	June 2016	50
COPD or asthma					
NCT02233114	Do Yogic Exercises (12 weeks) Increase Respiratory Function in Patients with Obstructive Lung Diseases?	Randomized, single-blind, parallel group study	Secondary	December 2017	40
Chronic respiratory insufficiency					
NCT01458314	Non Invasive Mechanical Ventilation in Chronic Respiratory Insufficiency Patients During Rehabilitation	Randomized, open-label, parallel group study	Secondary	June 2015	50
Chronic respiratory failure					
NCT00994552	Comparison of Pressure Support and Pressure Control Ventilation in Chronic Respiratory Failure	Randomized, single-blind crossover study	Secondary	April 2010	20
Congenital heart disease					
NCT02438293	The Impact of Rhinovirus Infections in Paediatric Cardiac Surgery’ (RISK)	Prospective, observational study	Secondary	June 2016	250
Intraventricular hemorrhage					
NCT02231411	Neonatal Resuscitation With Intact Cord (NRIC)	Randomized, single-blind, parallel group study	Secondary	August 2016	150
Lung cancer					
NCT02493114	Functional Status in Patients Undergoing Curative Treatment for Lung Cancer	Prospective, observational study	Secondary	November 2017	80
Mechanically ventilated patients					
NCT02003053	A Randomized, Controlled Trial of Inspiratory Muscle Training (IMT) in the ICU and CCU	Randomized, single-blind, parallel group study	Primary	September 2015	40
Obstructive sleep apnea					
NCT02259660	Airway Muscle Training for Obstructive Sleep Apnea (OSA)	Randomized, double-blind, parallel group study	Secondary	September 2016	60
Stroke					
NCT02400138	Home-based Respiratory Training After Stroke	Randomized, double-blind, parallel group study	Primary	November 2016	20

^a^Clinical trials in progress as of October 31^st^, 2015

*BiPAP* biphasic positive airway pressure, *CCU* critical care unit, *COPD* chronic obstructive pulmonary disease, *DMD* Duchenne muscular dystrophy, *FVC* forced vital capacity, *ICU* intensive care unit, *MIP* maximum inspiratory pressure, *OMT* osteopathic manipulative treatment, *PD* Parkinson’s disease, *TNI* therapy with nasal insufflation

### Relationship between MIP and survival in patients with NMD

As noted, respiratory failure is a common cause of premature death in patients with NMD [19]. Consequently, patients with progressive disease require frequent monitoring of their pulmonary function. Sensitive, noninvasive predictive measures are needed to quantify the risk of mortality due to respiratory failure in these patients; predictive measures could also quantify the potential mortality risk benefit of a therapeutic intervention.

Studies have investigated the correlation between MIP and survival in various conditions (Table 3) [37–51]. In regard to NMD, the majority of data comes from patients with ALS. All ALS published studies we identified consistently found a correlation between MIP and survival [37–41]. A cohort study in 95 patients with ALS found a significant association between MIP and 1-year survival (*P* < 0.05) [37]. The study found that, whereas a normal (>80% predicted) supine FVC predicted a > 80% chance of 1-year tracheostomy-free survival, a normal MIP or MEP predicted a > 90% chance of survival. In a second ALS study, reduced MIP predicted poor 2-years survival. Extensively controlled for nonpulmonary factors known to predict survival in ALS, Kaplan-Meier and receiver operating characteristic curve analysis showed that 2-years survival was more probable in patients with initially normal MIP values (*P* = 0.0001) compared with patients who had initially reduced MIP (<70 cm H_2_O; *P* < 0.05) [38]. In a third study of ALS patients (*N* = 21), MIP (−60 cm H_2_O or less) was 100% sensitive as a “threshold” for predicting 18-months survival, whereas FVC (<80% of predicted) was not as sensitive for predicting survival (<80% sensitive) [39]. In a fourth study of 53 patients with ALS, comparison of baseline data in patients who were dead or alive at 18 months showed that survivors had a higher mean MIP (38 ± 24% predicted) than nonsurvivors (20 ± 18% predicted; *P* < 0.01). The absence of cough spikes (defined as peak flow rate transients during voluntary cough) had no significant influence on survival [40]. Finally, clinical results from a 5-years prospective, comparative trial of patients with ALS using noninvasive ventilation found that determinants of respiratory function (including MIP [*P* = 0.0001]) were an independent predictor of 5-years survival, emphasizing the potential utility of MIP as a prognostic indicator in patients with ALS [41].

**Table 3:** Summary of studies investigating the correlation between MIP and survival

Reference	Therapy Area/Population	Study Type	N	MIP and Survival
Correlation between MIP and survival				
Schmidt EP et al., 2006 [37]	ALS	Cohort study	95	MIP was an important predictor of 1-year survival (*P* < 0.05) after controlling for nonpulmonary factors known to predict survival in ALS
Baumann F et al., 2010 [38]	ALS	Cohort study	80	MIP was significantly associated with survival (*P* < 0.05); survival time was increased in patients with normal MIP
Gay PC et al., 1991 [39]	ALS	Prospective study	21	MIP was 100% sensitive for predicting 18-months survival (*r* = 0.57; *P* < 0.007)
Chaudri MB et al., 2002 [40]	MND (ALS)	Single-center cohort study	53	Survivors had a higher percentage of predicted MIP than nonsurvivors (37.83 ± 24.32% vs 20.13 ± 18.43%; *P* < 0.01)
Lopes Almeida JP, et al., 2012 [41]	ALS	Prospective, comparative study	60	There was a significant correlation between MIP and 5-years survival (*P* = 0.02) in patients with ALS using NIV
Benzo R et al., 2013 [42]	COPD	Analysis of clinical data from NETT	1218	Decrease in MIP > 11 cm H_2_O was a predictor of 1-year mortality (OR, 2.19; *P* = 0.0217)
Gray-Donald K et al., 1996 [43]	COPD	3 to 5-years follow-up of a double-blind RCT	348	Low MIP was a significant independent predictor of respiratory (HR, 0.64; 95% CI, 0.44–0.95) and all-cause mortality (HR, 0.67; 95% CI, 0.47–0.95)
Schols AMWJ et al., 1998 [44]	COPD	RCT	203	Improvement in MIP during rehabilitation decreased the risk of death
Hodgev VA et al., 2006 [45]	COPD	Prospective cohort study	63	A Cox proportional hazards analysis showed that MIP was a significant predictor of mortality (*r* = 0.91; 95% CI, 0.85–0.97; *P* = 0.005)
Meyer FJ et al., 2001 [47]	Heart failure	Prospective study	244	In a univariate Cox regression analysis, MIP was found to be a significant prognostic indicator of survival (*P* = 0.001)
Frankenstein L et al., 2009 [48]	Heart failure	Prospective, observational study	686	MIP was identified as a significant predictor of survival by univariate analysis; survivors had a significantly higher MIP and percentage of predicted MIP than nonsurvivors
Ionescu AA et al., 1998 [49]	Cystic fibrosis	Single-center study	49	Mean % predicted MIP (SD) for survivors was 85.5% (28.4) compared with 64.1% (23.9) for nonsurvivors
Marroni CA et al., 2014 [50]	Liver cirrhosis	Prospective cohort study	86	Sixty-two percent of patients with MIP < −70 cm H_2_O survived compared with 93% of patients with MIP > −70 cm H_2_O (*P* = 0.0001)
Budweiser S et al., 2007 [46]	Chronic hypercapnic respiratory failure	Cohort study	464	MIP was a significant predictor of long-term survival; according to stepwise multivariate Cox regression analysis, P_0.1_/MIP was identified as an independent predictor of survival (*P* < 0.05)
van der Palen J et al., 2004 [51]	Elderly with a mean age of 72.5 years	Cohort study	3839	Subjects in the lowest quintile of MIP had a 1.5-fold increased risk of cardiovascular-related death (HR, 1.54; 95% CI, 1.09–2.15) after adjustment for nonpulmonary function covariates
No correlation between MIP and survival				
Nizet TAC et al., 2005 [58]	Chronic hypercapnic COPD	Prospective, single-center study	47	No significant association
Frankenstein L et al., 2008 [54]	CHF	Prospective, single-arm study	158	No significant association
Habedank D et al., 2013 [55]	CHF	Single-center study	249	No significant association
Hui D et al., 2014 [56]	Advanced cancer	Prospective, single-center study	222	No significant association
White AC et al., 2005 [59]	Hematopoietic stem cell transplantation	Prospective observational study	56	No significant association
Jackson M et al., 1994 [57]	Patients with a thoracoplasty for tuberculosis	Single-center study	32	No significant association

*ALS* amyotrophic lateral sclerosis, *CHF* congestive heart failure, *CI* confidence interval, *COPD* chronic obstructive pulmonary disease, *HR* hazard ratio, *MIP* maximum inspiratory pressure, *MND* motor neurone disease, *NETT* National Emphysema Treatment Trial, *NIV* noninvasive ventilation, *OR* odds ratio, *P*_*0.1*_ mouth occlusion pressure, *RCT* randomized controlled trial, *SD* standard deviation

In a further study recently published by one of the current authors [52], multiple outcome measures were obtained in 78 patients with ALS who were then followed until death. Low values for MIP were highly specific predictors of time to death or initiation of NIV; conversely, while VC was also a specific predictor, the cut points suggested by ROC analysis were >80% of normal at all time points except for 3 months mortality prediction (when it was 78%), suggesting that a normal VC was of limited practical value. However, a small (*N* = 18) study of MIP and survival in patients with Duchenne muscular dystrophy [53] did not find a predictive association. It should be noted that participants had extremely low values of MIP and VC at the start of study and that the analysis did not include the use of noninvasive ventilation.

Some studies in non-neuromuscular diseases—chronic obstructive pulmonary disease (COPD) [42–45], cystic fibrosis [49], liver cirrhosis [50], hypercapnic respiratory failure [46], and congestive heart failure [47, 48, 51]—suggest that MIP may correlate with survival, while others report no correlation [54–59] (Table 3), indicating that further investigation is needed. We also caution that in non-neuromuscular disease states, a reduced MIP may simply reflect generalized cachexia, which is a recognized marker of a poor prognosis and hyperinflation [60] in pulmonary disease.

### Relationship between MIP and QoL in patients with NMD

As NMD pathology progresses and patients develop respiratory dysfunction, QoL (with respect to a patient’s physical, emotional, social functioning, mental health, bodily pain, endurance, and general health perceptions) and sleep can be severely impacted [18, 61, 62]. The relationship between changes in MIP and QoL in NMD was addressed in 2 studies from our literature search: one in ALS [18] and one in patients with post-poliomyelitis syndrome [63].

Bourke et al. evaluated the impact of noninvasive intervention on QoL in 22 patients with ALS using the 36-Item Short Form (SF-36) and the National Center for Health Statistics General Well-Being Schedule and concluded that respiratory muscle weakness had an impact on QoL [18]. Overall, the researchers found that patients with ALS with significantly lower QoL displayed lower MIP values. Lower MIP values corresponded with lower SF-36 scores in all domains except the pain and physical components [18]. Instruments specifically assessing respiratory and sleep-related problems (eg, the Epworth Sleepiness Scale [ESS]) were most sensitive to changes in MIP [18]. In this regard, ESS scores were highest (indicating sleep disruption) in patients with MIP values below 50% and were lowest in patients with MIP values above 50% (10.3 and 4.8%, respectively; *P* = 0.01) [18].

Similar to the findings of Bourke et al., a cross-sectional study of 52 patients with post-poliomyelitis syndrome observed a significant correlation between MIP and both the fatigue severity scale and the Multidimensional Fatigue Inventory (MFI) (*r* = −0.31 and *r* = −0.41, respectively; *P* < 0.05). The researchers also found that a 10-unit decline for MIP (% predicted) corresponded to patients scoring a 0.3-unit increase on the General Fatigue dimension of the MFI scale [63]. This dimension of the MFI scale ranges from 4 to 20, with higher scores indicating more severe fatigue.

### Relationship between MIP and spirometry in patients with NMD

In patients with NMD whose lungs are restricted from fully expanding, spirometry is widely used to assess respiratory muscle function. Patients performing spirometry are asked to take a maximal inspiration and perform a FVC maneuver. A drop of more than 20% of FVC going from the upright to the supine position is a useful diagnostic of diaphragmatic weakness [64]. FVC, however, has a curvilinear relationship with respiratory muscle strength, and substantial weakness may be present while FVC is still within the normal range [65].

Lung volume measurements are sometimes performed in patients with NMD, including TLC and functional residual capacity (FRC; defined as the amount of air in the lungs following normal expiration). In these patients, when inspiratory muscles are weak, then a maximum effort may be insufficient to fully expand the lungs, and the TLC and FRC will be reduced [22, 24]. Similarly, if abdominal muscle strength is impaired, residual volume may also be elevated [22].

When carbon monoxide gas transfer is measured, the classic picture of respiratory muscle weakness is a low diffusion capacity (DL_CO_) and an elevated transfer coefficient (K_CO_). However, a study in patients with NMDs showed that the rise in K_CO_ was often less than expected; in patients with combined inspiratory and expiratory muscle weakness, a reduced value was observed [66]. The results demonstrated the limitations of using K_CO_ in the diagnosis of respiratory muscle weakness. Gas exchange anomalies can also be multifactorial in their origin, for example, mechanical problems and airway obstructions can affect results [67].

While the above discussed spirometry tests have utility, they can be considered insensitive measures of respiratory muscle function since a significant reduction in lung volume may not be observed until severe impairment of respiratory muscles has occurred [67]. Also, other factors such as airway obstruction due to asthma or lower airway obstruction may affect the reliability of some spirometry results [5, 68–70]. Consistent with these limitations, when measured within 30 days of the need for tracheostomy in a clinical trial of therapeutics for ALS [71], VC was ≥ 60% predicted in 14% of ALS patients (*n* = 50).

MIP has physiological relationships with spirometry endpoints [65]. Specifically, in patients with a NMD, several studies have shown a correlation between MIP and FVC. In a study of patients with Duchenne muscular dystrophy, a significant 1-year decrease in MIP was associated with decreases in FVC, FEV_1_ (defined as forced expiratory volume in 1 sec), and peak expiratory flow rate (*P* < 0.05 for all measures) [72]. Similarly, Schmidt et al. found a significant association between MIP and upright FVC in a 1-year cohort study of 95 patients with ALS [37].

Also, the LOTS study investigated the effect of alglucosidase alfa treatment in patients with late-onset Pompe disease [73]. Patients who entered an open-label extension phase of this trial showed additional improvement in MIP but a slight decline in FVC from week 78 through week 104; a statistical correlation however was not reported [74]. Finally, a RCT conducted by Cheah et al. to assess the effects of a 12-weeks inspiratory muscle training program in patients with ALS found that improvements in MIP reflected improvements in FVC and TLC [75]. In 3 RCTs where MIP improvements were observed following pharmacologic treatment in patients with non-neuromuscular diseases, these values also correlated with improvements in other pulmonary measures, including FVC (as well as airway resistance and MEP) [26, 30, 31].

Additional non-RCT studies supporting a correlation between MIP and spirometry in patients with NMDs have also been performed in patients with Pompe disease, Guillain-Barré syndrome, and myasthenia gravis [70, 74, 76, 77]. In a prospective cohort study in patients with Pompe disease, MIP and MEP were both strongly correlated with VC (*r* = 0.75 and *r* = 0.79, respectively) [76]. Follow-up data (median 1.6 years) showed that VC (upright or supine) deteriorated by 0.9–1.2% points per year, respectively, with deteriorations in MIP and MEP of 3.2% (*P* = 0.018) and 3.8% (*P* < 0.01) per year, respectively [76]. In a study of patients with respiratory muscle weakness and one study of multiple NMDs, MIP was found to significantly correlate with FRC (*r* = 0.62; *P* < 0.001) and VC (*r* = 0.88; *P* < 0.001) [77]. A direct investigation of the sensitivity of MIP versus VC in patients with either Guillain-Barré syndrome (*n* = 40) or myasthenia gravis (*n* = 44) found a linear relationship between the 2 measurements [70].

Taken together, these studies demonstrate that in patients with NMD there is a correlation between MIP and FVC, FEV_1_, body plethysmography, and diffusion techniques. In addition, MIP could have utility as a clinical endpoint in therapeutic trials for the treatment of neuromuscular diseases. A recent study in ALS patients with progressive respiratory dysfunction provided indirect evidence that reductions in MIP occur prior to reductions in FVC [28]. In patients with ALS, clinicians detected the progression of respiratory dysfunction 6.5 months earlier when monitoring MIP compared with FVC alone. This indicates that MIP may be a more sensitive measure of respiratory disease progression, supporting its potential utility as a clinical trial endpoint.

### Relationship between MIP and nonpulmonary measures in patients with NMD

Walking tests are an integrated assessment of cardiac, pulmonary, circulatory, and muscular capacity, providing a measure of the functional exercise level required to undertake daily physical activities. Neuromuscular and pulmonary studies investigating the impact of MIP on ambulatory measures showed that, in some cases, improvements in MIP coincided with improvements in walking tests [25, 34, 54, 74, 75, 78–85]. Two studies were identified comparing MIP with walking tests: one in Pompe disease and one in ALS. The study of patients with Pompe disease [74] receiving enzyme replacement therapy found that changes in the 6-min walk test (6MWT) were directionally consistent with changes in MIP. However, no such association was found in the study of patients with ALS [75].

## Discussion

Since the development of respiratory failure is a significant predictor of early death, many clinical trials currently employ established spirometry endpoints, including FVC, to evaluate an intervention in patients with NMD. However, as these endpoints are measures of overall pulmonary function, they may also be affected by factors that are independent of respiratory muscle dysfunction. Given that respiratory muscle dysfunction is common in neuromuscular diseases, directly evaluating diaphragm muscle strength by measuring MIP could complement spirometric endpoints in studies of patients with these diseases. In this regard, our analysis identified the use of MIP as an endpoint in several RCTs of pharmacologic therapies across a spectrum of diseases [26, 30–36]. These trials found MIP to be a clinically relevant outcome measure in chronic diseases when respiratory failure is secondary to respiratory muscle weakness.

However, diminished MIP does not always reliably confirm inspiratory muscle weakness. This is due to MIP measurement errors, including submaximal effort, poor transmission of intrathoracic pressure to the extrathoracic airways [22] as well as NMD patient-device interface issues or additional chest wall alterations [75]. For example, interface issues occur in patients with NMD with bulbar and/or facial weakness who have difficulty making a good lip seal. However, with proper training, MIP can be a reliable, accurate, and an early indicator of respiratory muscle weakness, which is more independent of existing lung abnormalities than FVC and VC [22, 86–88]. In this respect, a number of ongoing and planned clinical trials are evaluating MIP as a primary or secondary endpoint in studies of patients with NMDs (Table 2).

In order to further validate MIP as a clinical endpoint in studies of patients with NMD, it is important to establish whether MIP is associated with clinically meaningful outcomes, such as time to ventilator support or even overall survival. In this regard, our analysis found that MIP was correlated with survival in patients with some NMDs (particularly ALS) and that MIP made an important contribution to predictive multivariate modeling analyses [37–41]. Studies in patients with ALS consistently found that higher MIP values were associated with increased survival. One study found MIP to be 100% sensitive as a threshold for predicting survival in patients with ALS at 18-months follow-up [39]. Additionally, clinical studies that extended up to 5 years found a positive association between MIP and survival in a number of therapy areas [41, 43].

Our analysis also suggests that MIP may be reflective of challenges faced by patients with NMD in their daily life. Two studies conducted in patients with NMD found that MIP correlated with improvements in QoL scores, including domains relating to sleep and fatigue [18, 63]. Another study in patients with Pompe disease indicated that MIP was significantly correlated with the 6MWT [74, 85]. These findings suggest that MIP may have useful long-term clinical relevance in patients with NMDs. Furthermore, in the majority of NMD studies examined, there was a strong correlation between MIP with other pulmonary measures, including with FVC, FEV_1_, VC, and TLC [37, 72, 74–76]. In addition, there is some evidence to indicate that, when respiratory muscle function starts to deteriorate, MIP measurements may decrease earlier than other pulmonary measures, suggesting that MIP may be a more sensitive method for monitoring patients [28]. In ALS patients, for example, reduced inspiratory muscle strength (MIP) was noted after cessation of inspiratory muscle training despite other pulmonary measures remaining unchanged [75].

In addition to MIP, the SNIP test can be used to measure respiratory muscle strength in the clinic. Like MIP, SNIP also reflects esophageal pressure in most patients, but a minority of patients will obtain substantially larger values on one test than the other [89]. SNIP is measured by plugging one nostril while the other is free; this test is useful when there is facial weakness or dental malocclusion, which may make the MIP test difficult [5]. It is a reproducible and accurate measure of inspiratory muscle strength [90] and can be reliably performed and used even in infants and children [91]. SNIP has been used to monitor respiratory muscle strength in patients with NMD [92, 93]. However, it has limitations such as underestimating esophageal pressure swing in patients with severe nasal obstruction or airway diseases such as COPD, which impair pressure transmission [94–96]. Additionally, SNIP measures a fast contraction of inspiratory muscles, whereas MIP measures a sustained isometric contraction [95]. As isometric muscle force of limb muscles is a standard measure of muscle function, it has been argued that in some NMDs, such as ALS, MIP might provide a more meaningful measurement of respiratory muscle function than SNIP [95].

Our analysis of MIP as a clinical outcome measure in patients with NMDs is limited by the small number of published studies and small sample sizes. However, the fact that there are currently 12 registered trials in patients with NMD where MIP is being used as a clinical endpoint suggests that specific measurements of ventilatory function are important outcomes in these patient cohorts. These trials will provide further evidence of the reliability and utility of MIP. Additionally, MIP may prove to be more sensitive than FVC for assessing ventilatory dysfunction since spirometric pulmonary function tests are influenced by other factors (including scoliosis and other lung diseases), which may affect the reliability of results [5, 68–70].

## Conclusions

In summary, our analysis supports the use of MIP as a diagnostic of respiratory muscle dysfunction in patients with chronic NMDs and its utility as an endpoint in future clinical trials which monitor the efficacy of therapeutics in neuromuscular diseases. Through continued investigation of MIP in NMD, clinicians and researchers will gain a comprehensive understanding of the role of this direct measure of respiratory muscle strength in clinical practice.

## Abbreviations

6MWT: 6-minute walk test
ALS: Amyotrophic lateral sclerosis
COPD: Chronic obstructive pulmonary disease
ESS: Epworth sleepiness scale
FEV_1_: Forced expiratory volume in 1 second
FRC: Functional residual capacity
FVC: Forced vital capacity
MEP: Maximal expiratory pressure
MFI: Multidimensional fatigue inventory
MIP: Maximal inspiratory pressure
NMD: Neuromuscular disease
QoL: Quality of life
RCT: Randomized controlled trial
SF-36: 36-Item short form
SNIP: Sniff nasal inspiratory pressure
TLC: Total lung capacity
VC: Vital capacity
